# Artificial intelligence-assisted assessment of metabolic response to tebentafusp in metastatic uveal melanoma: a long axial field-of-view [^18^F]FDG PET/CT study

**DOI:** 10.1007/s00259-025-07504-8

**Published:** 2025-09-06

**Authors:** Christos Sachpekidis, Devayani Machiraju, Dimitrios Stefanos Strauss, Leyun Pan, Annette Kopp-Schneider, Lars Edenbrandt, Antonia Dimitrakopoulou-Strauss, Jessica C. Hassel

**Affiliations:** 1 Clinical Cooperation Unit Nuclear Medicine, German Cancer Research Center (DKFZ), Im Neuenheimer Feld 280, 69210 Heidelberg, Germany; 2 Department of Dermatology and National Center for Tumor Diseases (NCT), University Hospital Heidelberg, Heidelberg, Germany; 3 Department of Diagnostic and Interventional Radiology, University Hospital Heidelberg, Heidelberg, Germany; 4 Division of Biostatistics, German Cancer Research Center (DKFZ), Heidelberg, Germany; 5 Department of Clinical Physiology, Region Västra Götaland, Sahlgrenska University Hospital, Gothenburg, Sweden; 6 Department of Molecular and Clinical Medicine, Institute of Medicine, Sahlgrenska Academy, University of Gothenburg, Gothenburg, Sweden

**Keywords:** Metastatic uveal melanoma, Tebentafusp, [^18^F]FDG LAFOV PET/CT, Deep learning, Artificial intelligence, Total metabolic tumor volume (TMTV), Total lesion glycolysis (TLG), PERCIST, Treatment response evaluation, CtDNA

## Abstract

**Purpose:**

Tebentafusp has emerged as the first systemic therapy to significantly prolong survival in treatment-naïve HLA-A*02:01 + patients with unresectable or metastatic uveal melanoma (mUM). Notably, a survival benefit has been observed even in the absence of radiographic response. This study aims to investigate the feasibility and prognostic value of artificial intelligence (AI)-assisted quantification and metabolic response assessment of [^18^F]FDG long axial field-of-view (LAFOV) PET/CT in mUM patients undergoing tebentafusp therapy.

**Materials and methods:**

Fifteen patients with mUM treated with tebentafusp underwent [^18^F]FDG LAFOV PET/CT at baseline and 3 months post-treatment. Total metabolic tumor volume (TMTV) and total lesion glycolysis (TLG) were quantified using a deep learning-based segmentation tool On the RECOMIA platform. Metabolic response was assessed according to AI-assisted PERCIST 1.0 criteria. Associations between PET-derived parameters and overall survival (OS) were evaluated using Kaplan–Meier survival analysis.

**Results:**

The median follow up (95% CI) was 14.1 months (12.9 months – not available). Automated TMTV and TLG measurements were successfully obtained in all patients. Elevated baseline TMTV and TLG were significantly associated with shorter OS (TMTV: 16.9 vs. 27.2 months; TLG: 16.9 vs. 27.2 months; *p* < 0.05). Similarly, higher TMTV and TLG at 3 months post-treatment predicted poorer survival outcomes (TMTV: 14.3 vs. 24.5 months; TLG: 14.3 vs. 24.5 months; *p* < 0.05). AI-assisted PERCIST response evaluation identified six patients with disease control (complete metabolic response, partial metabolic response, stable metabolic disease) and nine with progressive metabolic disease. A trend toward improved OS was observed in patients with disease control (24.5 vs. 14.6 months, *p* = 0.08). Circulating tumor DNA (ctDNA) levels based on GNAQ and GNA11 mutations were available in 8 patients; after 3 months Of tebentafusp treatment, 5 showed reduced Or stable ctDNA levels, and 3 showed an increase (median OS: 24.5 vs. 3.3 months; *p* = 0.13). Patients with increasing ctDNA levels exhibited significantly higher TMTV and TLG on follow-up imaging.

**Conclusion:**

AI-assisted whole-body quantification of [1⁸F]FDG PET/CT and PERCIST-based response assessment are feasible and hold prognostic significance in tebentafusp-treated mUM. TMTV and TLG may serve as non-invasive imaging biomarkers for risk stratification and treatment monitoring in this malignancy.

## Introduction

Uveal melanoma (UM) is a rare malignancy originating from melanocytes within the uveal tract [1]. While local treatment of the primary tumor is usually successful, nearly half of patients eventually develop metastatic disease (mUM), predominantly to the liver [2]. Historically, mUM has carried a dismal prognosis, with median overall survival (OS) Of approximately 1 year [3, 4]. However, the advent of tebentafusp has altered the therapeutic landscape, as the first systemic therapy to show a significant survival benefit in previously untreated, HLA-A*02:01-positive adults with unresectable or metastatic UM [5–7].

Tebentafusp is a first-in-class immune-mobilizing monoclonal T-cell receptor against cancer (ImmTAC) bispecific protein that binds to a specific peptide from the melanoma protein gp100 presented by HLA-A ∗ 02:01 on the surface of a tumor cell and to CD3 on the surface of a T cell, inducing polyclonal T cell activation and redirected killing of melanoma cells [8, 9]. In the IMCgp100-202 phase 3 trial, tebentafusp significantly improved OS compared with investigator’s choice of treatment (pembrolizumab, ipilimumab, or dacarbazine), with a median OS Of 21.6 months vs 16.9 months, and a 1-year OS rate Of 73% vs 59% [5]. The 3-year analysis confirmed a continued survival benefit, showing a 9% advantage [7]. Notably, benefit was observed even in patients whose best response was progressive disease (PD), suggesting that tebentafusp may affect the tumor microenvironment in ways not captured by conventional imaging.

[^18^F]FDG PET/CT is an established imaging modality in advanced cutaneous melanoma, recommended for staging, restaging, and treatment monitoring in AJCC Stage III–IV disease [10, 11]. Emerging data support its utility in the diagnosis and prognostication of mUM [12, 13]. Recent advances, especially the development of long axial field of view (LAFOV) PET/CT systems, have significantly improved sensitivity and image quality, enabling shorter acquisition times and low-dose protocols that may benefit melanoma imaging [14].

Conventional PET evaluation relies on manual segmentation and analysis of volumetric features from volumes of interest (VOIs), yielding metrics like standardized uptake value (SUV), total metabolic tumor volume (TMTV), and total lesion glycolysis (TLG). However, manual methods are time-consuming, prone to variability and bias, and compromise the reproducibility and accuracy of quantitative assessments. These limitations can affect diagnostic precision and response evaluation. Automated segmentation, particularly deep learning-based approaches, offer a promising solution by enabling rapid, consistent, and objective whole body VOI delineation, improving workflow and reliability of tumor quantification [15–19].

The aim of this study is to evaluate the performance of a novel three-dimensional deep learning-based tool for the automated quantification of tumor burden on [^18^F]FDG LAFOV PET/CT images, in the context of treatment response monitoring in mUM patients receiving tebentafusp.

## Materials and methods

### Patients

Fifteen patients with mUM undergoing tebentafusp therapy were enrolled in this analysis. Patients received intravenous tebentafusp at a dose Of 20 μg On day 1, 30 μg On day 8, and 68 μg weekly thereafter. Patients were monitored Overnight after treatment for the first 3 weeks during dose escalation. Patients gave written informed consent to participate in the study and to have their medical records released. The study was conducted in accordance with the Declaration of Helsinki principles, with institutional approval by the ethical committee of the University of Heidelberg (S-826/2024). The patients’ characteristics are summarized in Table 1.

**Table 1:** Patient characteristics

Patient number	Age	Gender	Sites of metastatic disease on baseline PET/CT (visual analysis)	LDH level at baseline	LDH level at follow-up	Tebentafusp as systemic line of treatment	Previous treatment(s)
1	54	F	Hepatic and extrahepatic (nodal, lung, bone)	normal	elevated	Third line	Pembrolizumab, ipilimuab/nivolumab, stereotactic radiosurgery
2	61	F	Hepatic and extrahepatic (nodal)	elevated	elevated	First line	None
3	74	M	Hepatic	normal	normal	First line	None
4	57	F	Hepatic and extrahepatic (nodal, bone, adrenal)	elevated	elevated	Second line	Pembrolizumab, chemosaturation
5	60	F	Hepatic and extrahepatic (soft tissue)	normal	normal	Second line	Ipilimuab/nivolumab, radiotherapy
6	65	F	Hepatic	normal	normal	First line	None
7	61	F	Hepatic and extrahepatic (nodal, lung, bone, soft tissue)	normal	normal	First line	None
8	72	M	Hepatic	normal	normal	First line	None
9	69	M	Hepatic and extrahepatic (bone)	normal	normal	Second line	Pembrolizumab
10	79	F	Hepatic	normal	normal	First line	None
11	66	M	Hepatic	normal	normal	First line	None
12	70	F	-	elevated	normal	First line	None
13	58	M	Hepatic and extrahepatic (nodal, lung, bone, soft tissue, intestine, peritoneum)	elevated	elevated	First line	Chemosaturation
14	62	M	Hepatic and extrahepatic (bone)	elevated	elevated	First line	None
15	74	F	Hepatic	normal	normal	First line	None

*F* female; *M* male; *LDH* lactate dehydrogenase

### Detection of circulating tumor DNA (ctDNA)

ctDNA from plasma samples was extracted as reported before [20]. In short, plasma DNA was extracted using the QIAamp MinElute ccfDNA Mini Kit (catalog no. 55204, Qiagen) following the manufacturer’s protocol. DNA was eluted and quantified using the Qubit® 2.0 Fluorometer (Life Technologies). We used the Bio-Rad QX200 ddPCR system (Bio-Rad, Hercules, CA). OncobitTM PM, which consists of positive DNA controls and digital PCR assays targeting GNAQ209P, GNAQ209L, and GNA11209L mutations, was kindly provided by Oncobit AG (Switzerland). Control and primer sequences are proprietary to the company. Data was processed using QuantaSoft v.1.6 (Bio-Rad) and OncobitTM PM Analyzer.

### PET/CT data acquisition

[^18^F]FDG PET/CT was performed prior to treatment initiation (baseline PET/CT) and at 3 months following tebentafusp administration (follow-up PET/CT) in all patients. Participants fasted for at least 6 h prior to intravenous injection of a body weight–adjusted dose Of 2.0 MBq/kg [^1^⁸F]FDG. PET/CT imaging was conducted using a Biograph Vision Quadra scanner (Siemens Healthineers, Erlangen, Germany) 60 min post-injection (p.i.). Continuous whole-body imaging from skull to feet, was acquired Over 15 min. A low-dose CT scan (120 kV, 30 mA) was utilized for attenuation correction and anatomical co-registration. PET data were acquired with the new ultrahigh sensitivity (UHS) reconstruction mode using the full acceptance angle [maximum ring difference (MRD) 322], were attenuation-corrected, and reconstructed using a 440 × 440 matrix. Images were reconstructed with the manufacturer's standard algorithm, incorporating point spread function (PSF) and time-of-flight (TOF) modeling (4 iterations × 5 subsets), without Gaussian filtering, into 1.65 × 1.65 × 1.65 mm^3^ voxels.

### PET/CT data analysis

#### Visual analysis

PET/CT images were analyzed using an Aycan workstation and jointly reviewed by two experienced nuclear medicine physicians in melanoma imaging (CS, ADS). For visual interpretation, lesions were classified as [^1^⁸F]FDG-avid if they exhibited focal tracer uptake markedly exceeding background activity, irrespective of the presence of a corresponding anatomical abnormality on CT, and if the uptake pattern was consistent with metastatic disease.

#### AI-assisted image analysis and quantification

Automated [^18^F]FDG PET/CT image analysis, including segmentation, quantification and response evaluation to tebentafusp, was performed according to Positron Emission Tomography Response Criteria in Solid Tumors (PERCIST) 1.0 [21]using the cloud‐based RECOMIA platform (www.recomia.org) [22]. The platform’s digital PERCIST module incorporates AI to support the image reader.

The AI tool “Organ Finder” automatically segmented the liver and aorta, placing VOIs in optimal (most representative) locations within these organs to derive reliable threshold values [22]. Given the high hepatic tropism of mUM, thresholds were calculated using a cylindrical VOI (1 cm diameter, 2 cm axial length) centered in the descending thoracic aorta, defined as:

Threshold = 2 × SUV corrected for lean body mass (SULmean) + 2 × SD of the VOI [23].

Pixels with activity exceeding this threshold value were identified and segmented as indicative of metastatic involvement. Advanced post-processing steps were employed to minimize spillover effects from adjacent structures, accounting for the limited spatial resolution of PET. The AI algorithm was trained to exclude physiologic tracer uptake in organs such as the brain, heart, and urinary system.

Post-segmentation, the following quantitative, whole-body metrics were derived:

- TMTV (mL): volume of all segmented lesions.
- TLG (g): calculated as TLG = SUVmean × MTV.

#### AI-assisted PERCIST response assessment

Treatment response was assessed per PERCIST 1.0, by identifying the single lesion with the highest peak SUV corrected for lean body mass (SULpeak) exceeding the established threshold based On the descending thoracic aorta. A 1 cm^3^ VOI was placed over this lesion, with manual adjustments made by the interpreting physicians when necessary to ensure accurate lesions delineation.

The percentage change in SULpeak between baseline and follow-up PET/CT scans was calculated using the following formula:

$$\text{Percent Change}=100\times \left({\mathrm{FTL}}_{\mathrm{SULpeak}}-{\mathrm{BTL}}_{\mathrm{SULpeak}}\right)/{\mathrm{BTL}}_{\mathrm{SULpeak}}$$

where FTL_SULpeak_ refers to the SULpeak at follow-up, and BTL_SULpeak_ represents the baseline SULpeak.

Based on this calculation, treatment responses were categorized as follows:

- Complete metabolic response (CMR): SULpeak of the target lesion was below the threshold at follow-up.
- Partial metabolic response (PMR): a decrease in SULpeak of ≥ 30% and an absolute difference of ≥ 0.8 SUL units.
- Stable metabolic disease (SMD): a change in SULpeak (increase or decrease) of < 30%.
- Progressive metabolic disease (PMD): an increase in SULpeak of ≥ 30% and ≥ 0.8 SUL units, or the appearance of new lesions [21, 23].

Patients were subsequently grouped into two categories based on disease control rate (DCR):

- Disease control: Includes CMR, PMR, and SMD.
- No-disease control: Includes PMD.

### Statistical analysis

TMTV and TLG values exhibited a skewed distribution; therefore, results are presented as median and range values. To investigate the potential prognostic role of quantitative PET parameters (TMTV, TLG) and PERCIST-based metabolic response categories in relation to OS, Kaplan–Meier survival curves and the log-rank test were employed. OS for each PET/CT scan was defined as the time from the respective scan to either death or last follow-up. Median follow-up duration was estimated using the reverse Kaplan–Meier method. Statistical analysis was conducted using R (version 4.0.3, packages: survival, survminer, prodlim). Given the exploratory nature of the study, no correction for multiple testing was performed. Wilcoxon rank sum test was used for two-group comparisons of quantitative variables. The results were considered significant for *p*-values less than 0.05 (*p* < 0.05).

## Results

### Patient characteristics

A total Of 15 patients with mUM were included in this study (6 males, 9 females; mean age: 65 years). All patients received treatment with tebentafusp. The mean baseline serum lactate dehydrogenase (LDH) level was 331 U/l. Elevated LDH levels were Observed in 5 patients, while the remaining 10 patients had LDH values within the normal range. ctDNA levels at baseline and after three months Of treatment were available for 8 patients. Among these, 5 showed a reduction Or stabilization in ctDNA levels, whereas 3 exhibited an increase.

Visual reading of the baseline PET/CT scans revealed that 14 Out Of 15 patients had at least one [^1^⁸F]FDG-avid lesion indicative Of metastatic involvement, while One patient had no hypermetabolic lesions. Of the 14 patients with findings suggestive of metastases, six had hepatic involvement only, while eight had both hepatic and extrahepatic involvement. Notably, none of the patients who had received prior immune checkpoint inhibitors exhibited PET findings suggestive of immune-related adverse events. Detailed demographic and clinical characteristics are summarized in Table 1.

### AI-assisted [^18^F]FDG PET/CT image analysis

Using the predefined threshold for pathological tracer uptake, automatical calculation Of TMTV and TLG values was feasible for all patients at baseline and at 3 months following the initiation of tebentafusp treatment. The corresponding results are summarized in Table 2.

**Table 2:** The median values (range) Of the automated calculation Of TMTV and TLG values at start of treatment and after 3 months of tebentafusp

	Median TMTV (range), mL	Median TLG (range), g
Baseline PET/CT	44 (0—4473)	445 (0—24198)
Follow-up PET/CT	44 (0–3023)	434 (0–21007)

*TMTV* total metabolic tumor volume; *TLG* total lesion glycolysis

Patients with elevated LDH levels exhibited significantly higher TMTV and TLG values both at baseline (*p* < 0.01 for both TMTV and TLG) and follow-up (*p* = 0.01 for TMTV; *p* < 0.01 for TLG). compared to patients with normal LDH levels. Additionally, in the subgroup of patients with available ctDNA data at both time points (*n* = 8), individuals with an increase in ctDNA levels exhibited significantly higher TMTV and TLG values on follow-up imaging than those with stable or reduced ctDNA levels (*p* = 0.03 for both TMTV and TLG).

**Fig. 1:**
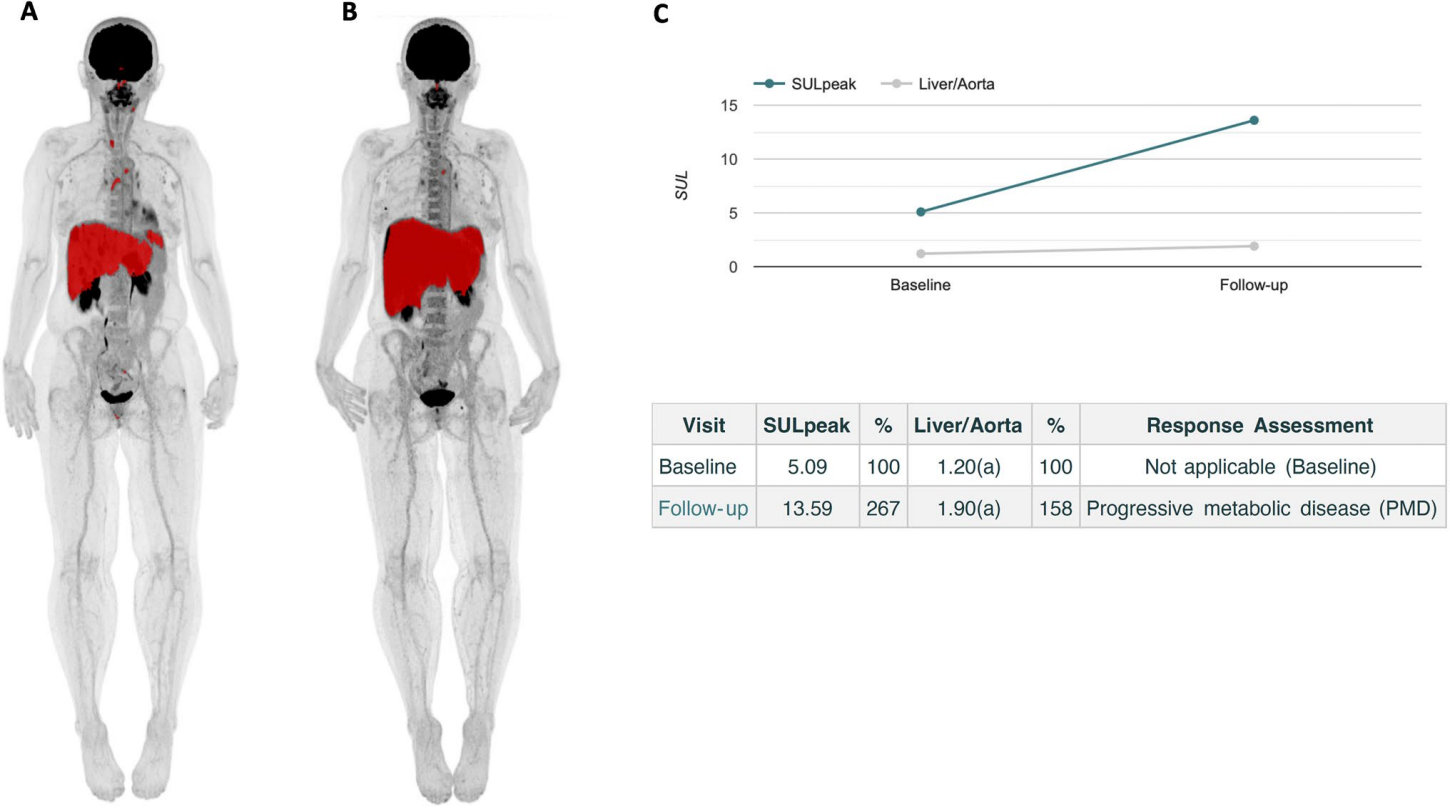
Representative example of AI-based automated calculation of TMTV and TLG in a mUM patient with diffuse liver involvement at baseline (**A**) and after 3 months of tebentafusp treatment (**B**). Both metabolic parameters increased markedly from baseline (TMTV = 739 ml, TLG = 2586 g) to follow-up (TMTV = 2046 ml, TLG = 13219 g). AI-assisted PERCIST analysis classified this case as progressive metabolic disease, as highlighted by the substantial rise in SULpeak of the target lesion (**C**)

**Fig. 2:**
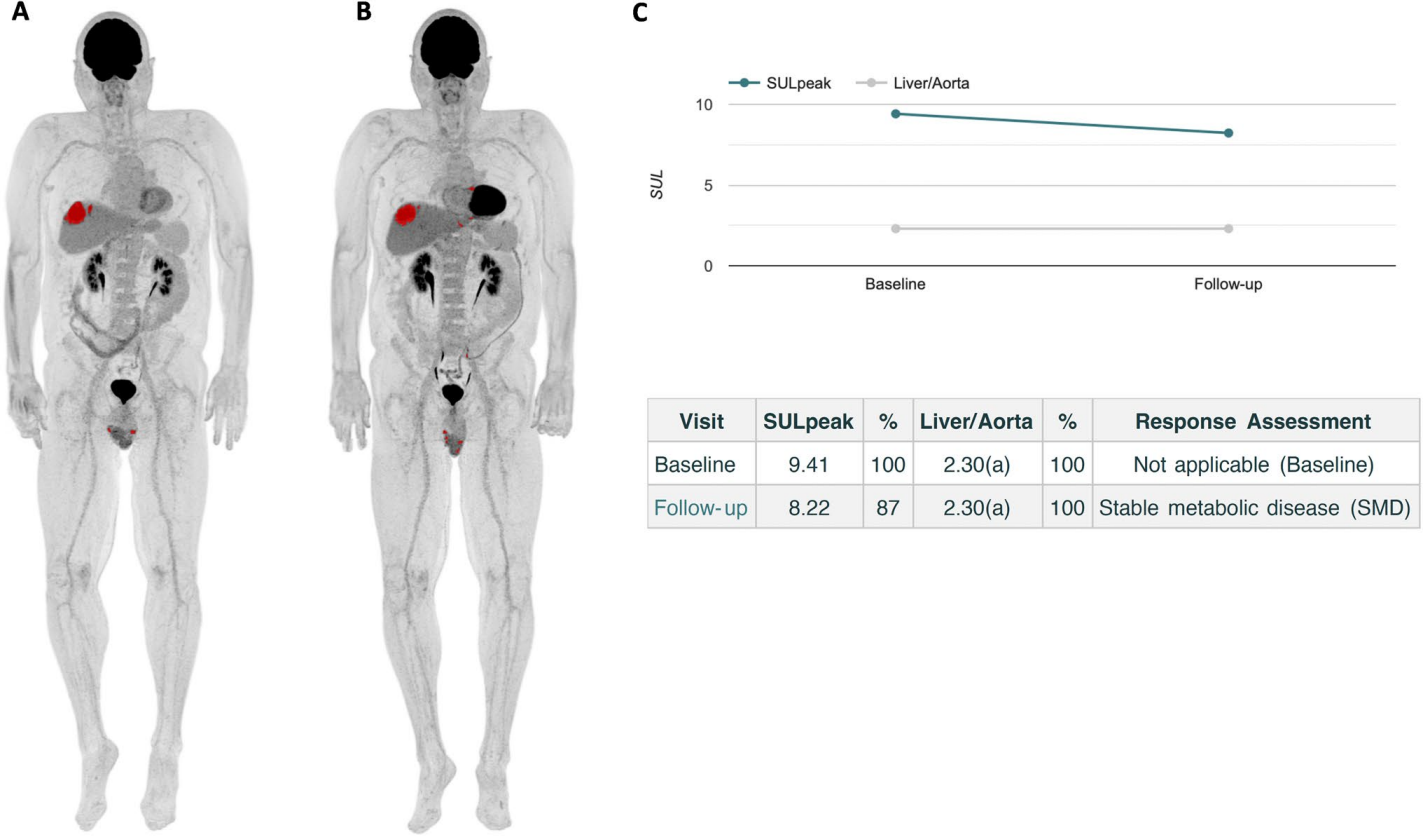
Representative example of AI-based automated whole-body TMTV and TLG quantification in a mUM patient at baseline (**A**) and after 3 months of tebentafusp treatment (**B**). Only minimal changes were observed in TMTV (baseline = 44 mL; follow-up = 44 mL) and TLG (baseline = 403 g; follow-up = 365 g). Consistent with these findings, AI-assisted PERCIST assessment indicated stable metabolic disease, as evidenced by minor fluctuations in the SULpeak of the target lesion (**C**)

Representative examples of AI-assisted TMTV and TLG quantification are presented Figs. 1 and 2.

### AI-assisted PERCIST response assessment

AI-assisted response assessment according to PERCIST 1.0 was feasible for all patients using follow-up PET/CT scans (Figs. 1 and 2). The distribution of metabolic responses was as follows:

- PMR: 1 patient
- SMD: 5 patients
- PMD: 9 patients

Based On DCR classification, 6 patients demonstrated disease control (CMR, PMR, or SMD), while 9 patients showed PMD.

### Survival analysis

The median follow-up duration from treatment initiation was 14.1 months (95% CI: 12.9–not available [NA]). At the time Of analysis 5 patients had died.

Patients with normal baseline LDH levels had a median OS Of 27.2 months (95% CI: NA–NA), compared to 17.1 months (95% CI: 6.7–NA) in patients with elevated LDH levels (*p* = 0.02). Regarding ctDNA, patients who showed a reduction Or stabilization in ctDNA levels had a median OS of 24.5 months (95% CI: 14.6–NA), whereas patients with increased ctDNA levels had a markedly shorter median OS Of 3.3 months (95% CI: 2.2–NA) (*p* = 0.13).

Patients with hepatic only involvement on baseline PET/CT had a median OS Of 27.2 months (NA–NA), while those with hepatic and extrahepatic involvement had a median OS Of 17.1 months (16.9–NA) (*p* = 0.09).

**Fig. 3:**
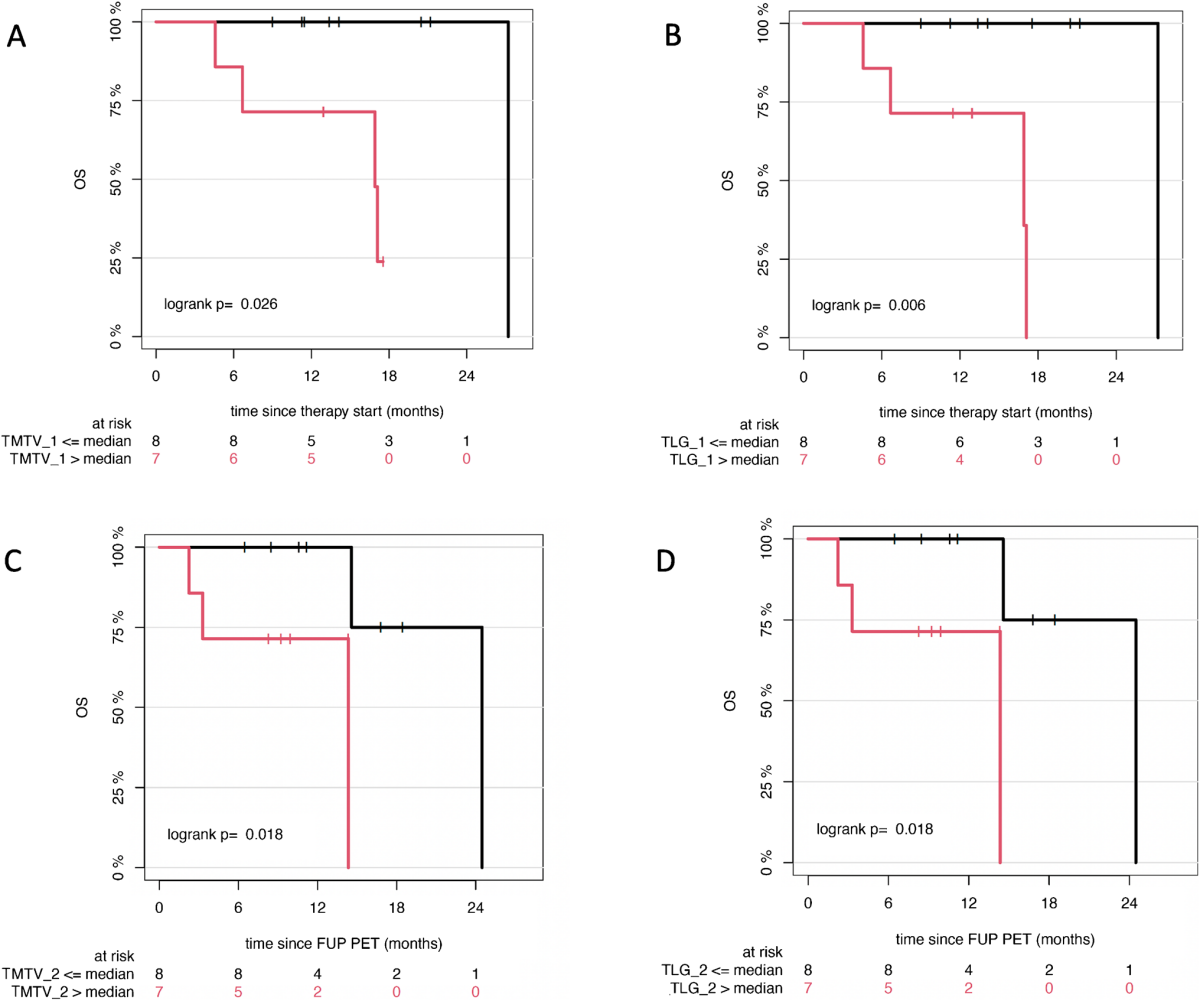
Kaplan–Meier estimates of OS according to AI-derived TMTV and TLG at baseline (**A**, **B**) and after 3 months of tebentafusp treatment (**C**, **D**). The number of patients at risk at each time point is indicated below each plot

In univariable analysis of automated volumetric whole-body PET/CT parameters, higher baseline TMTV and TLG were significantly associated with shorter OS. Similar associations were Observed for TMTV and TLG values obtained at 3 months post-treatment (Table 3) (Fig. 3).

**Table 3:** Prognostic significance of the AI-derived PET biomarkers TMTV and TLG for overall survival

	Median overall survival (95% CI)	
Baseline PET/CT		
TMTV	≤ median	27.2 (NA–NA)
	> median	16.9 (6.7–NA)
	*p*	0.03*
TLG	≤ median	27.2 (NA–NA)
	> median	16.9 (6.7–NA)
	*p*	< 0.01*
Follow-up PET/CT		
TMTV	≤ median	24.5 (14.6–NA)
	> median	14.3 (3.3–NA)
	*p*	0.02*
TLG	≤ median	24.5 (14.6–NA)
	> median	14.3 (3.3–NA)
	*p*	0.02*

*Statistically significant difference

*TMTV* total metabolic tumor volume; *TLG* total lesion glycolysis; 95% *CI* 95% confidence interval; *NA* not available

**Fig. 4:**
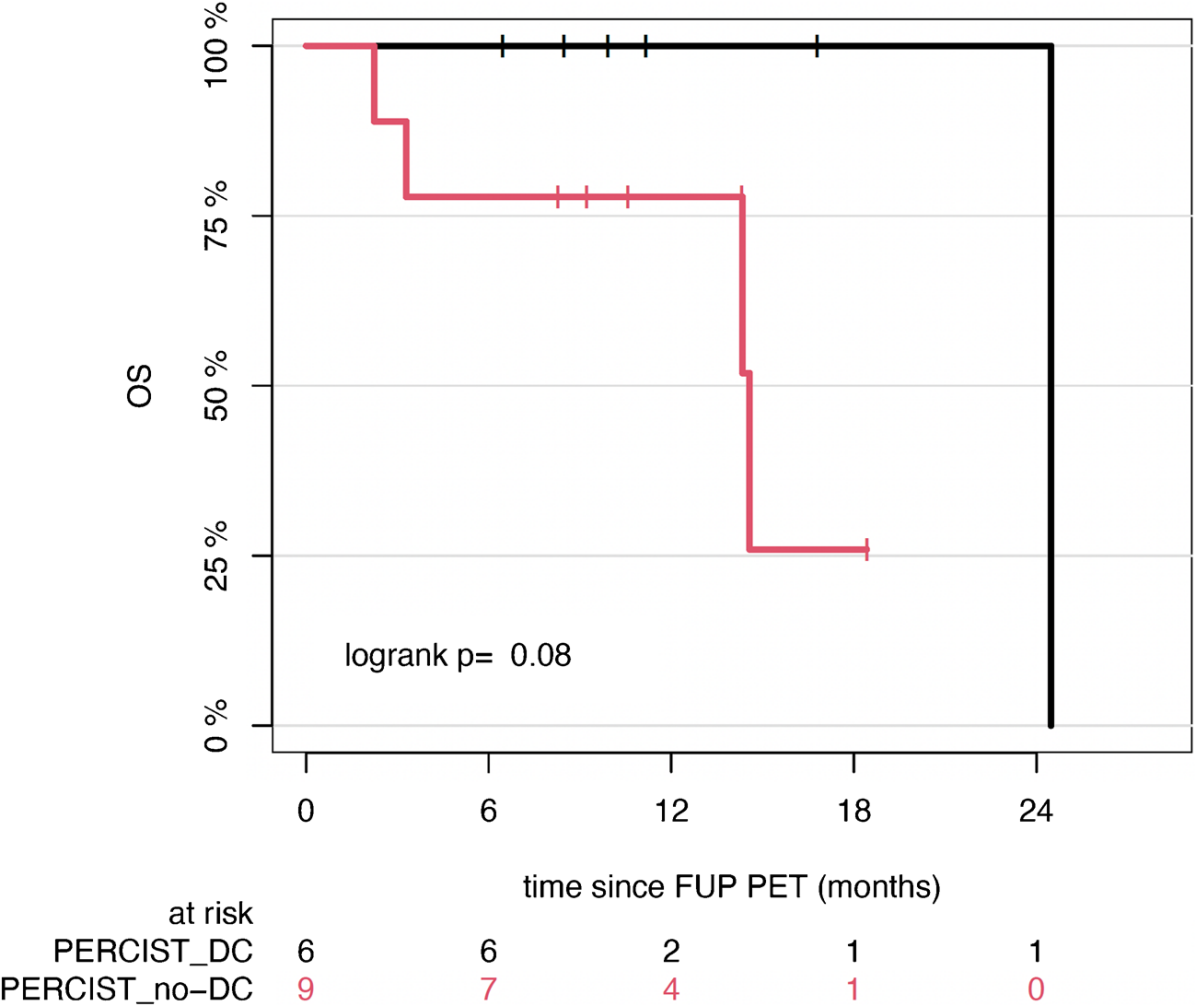
Kaplan–Meier estimates of OS according to AI-assisted PERCIST response categories. Patients were grouped by disease control (DC) versus no disease control (no-DC). The number of patients at risk for each group is shown below the survival curves

The application Of AI-assisted PERCIST revealed a trend toward improved OS among patients who achieved disease control, as defined by the DCR classification, compared to those with PMD. Patients classified as having disease control demonstrated a median OS of 24.5 months (95% CI: NA–NA), whereas those without disease control had a median OS Of 14.6 months (95% CI: 14.3–NA). Although the difference did not reach statistical significance, the trend was notable (*p* = 0.08) (Fig. 4).

## Discussion

This study is the first to evaluate AI-assisted quantification and metabolic response assessment using state-of-the-art LAFOV PET/CT imaging in mUM patients treated with the novel agent tebentafusp. Our key findings are threefold: First, automated, whole-body volumetric assessment of mUM tumor burden using PET/CT was feasible and yielded reliable measurements Of TMTV and TLG. Second, elevated AI-derived TMTV and TLG values, both at baseline and after 3 months of tebentafusp treatment, were significantly associated with worse patient outcomes. Third, AI-assisted application of PERCIST criteria for metabolic response assessment was feasible and revealed a trend toward improved OS in patients achieving disease control compared to those with progressive disease.

Tebentafusp represents a significant therapeutic breakthrough in the treatment of mUM, offering survival benefits in a disease historically resistant to systemic therapies, as highlighted in the pivotal IMCgp100-202 trial [5, 7]. Importantly, this trial highlighted a discordance between overall survival and conventional radiographic response by RECIST, underscoring the limitations of standard imaging criteria in capturing the full clinical benefit of tebentafusp. This disconnect emphasizes the need for alternative, reliable biomarkers that more accurately capture treatment efficacy and can guide clinical decision-making.

To address this, we utilized PET/CT, a functional imaging modality with more established utility in advanced cutaneous melanoma than mUM, complemented by AI-based tools for automated whole body tumor burden quantification. Building on methodologies used in our previous work [18, 24], we demonstrate that, following application of appropriate thresholds, the automated calculation of PET volumetric parameters is not only feasible but also correlates significantly with patient survival outcomes. These findings support the potential role of AI-assisted TMTV and TLG as prognostic biomarkers and as valuable tools for treatment monitoring in tebentafusp-treated mUM patients, where diffuse hepatic involvement often complicates manual lesion delineation. In this context, automated PET-based volumetric assessment provides a practical, efficient alternative. Our results align with recent studies that employ AI-driven segmentation of whole-body [^18^F]FDG PET/CT in advanced cutaneous melanoma —a biologically distinct tumor entity with different therapeutic options— where similarly disseminated metastatic patterns are observed. These approaches, leveraging automatically extracted features, may enable reliable quantification of metabolic biomarkers and potentially enhance risk stratification in patients undergoing immune checkpoint inhibitor therapy [19, 25–28].

Moreover, our exploratory analysis of ctDNA suggests a potential complementary role to PET/CT-derived biomarkers. Although not yet part of standard clinical practice in mUM, ctDNA dynamics have been associated with patient outcomes [7, 20] and, importantly, showed for the first time a correlation with TMTV and TLG values. Patients with increasing ctDNA levels exhibited significantly higher metabolic tumor burden under tebentafusp therapy, reinforcing the potential of combining molecular and imaging biomarkers to improve early response prediction and patient stratification. However, this analysis was limited by the availability of ctDNA data in only a subcohort of patients, warranting cautious interpretation.

Among PET-based response criteria, PERCIST offers a standardized, widely accepted framework for metabolic treatment evaluation, favored over EORTC criteria in clinical trials due to its rigorous definitions for lesion selection, region-of-interest classification, and response evaluation [22, 29]. Following the model Of RECIST, PERCIST 1.0 categorizes metabolic treatment response into four classes based on changes in SULpeak of the most metabolically active lesion between scans, using uptake thresholds derived from a reference organ—in our case, the descending thoracic aorta.

A key challenge in applying PERCIST to tumors with extensive metastatic involvement, such as mUM, lies in the consistent and accurate target lesion identification and delineation. To mitigate this, we utilized the RECOMIA platform’s digital PERCIST module, which integrates AI for automatic segmentation of reference organs and lesions, thereby enhancing the objectivity and reproducibility of response assessment. While AI-assisted PERCIST response assessment did not reach statistical significance in predicting OS, it demonstrated a promising trend favoring patients with disease control over those with progressive metabolic disease, suggesting complementary prognostic value alongside TMTV and TLG metrics. It is worth noting that the current RECOMIA platform does not automatically flag the appearance of new hypermetabolic lesions as a criterion for progressive disease, requiring manual oversight by interpreting physicians—though this limitation did not affect the present study.

A notable strength of our study is the application of a cutting-edge LAFOV PET/CT system, which offers increased sensitivity, superior lesion detectability, and the capability to reduce scan duration and administered radiotracer dose [30–32]. This allowed us to acquire high-quality, whole-body images within 15 min using a low [^18^F]FDG activity Of 2 MBq/kg. While this dose is below the 3.0 MBq/kg range recommended by the European Association of Nuclear Medicine (EANM) guidelines for conventional PET/CT [33], the ultra-high sensitivity Of the LAFOV system enabled excellent image quality and quantitative performance. Importantly, Our group has previously shown that a 50% reduction in both acquisition time and radiotracer dose in melanoma imaging can be achieved without compromising diagnostic accuracy [14]. Although lower injected activity could theoretically compromise quantification in very small or low-contrast lesions, no such limitation was observed in our dataset, likely due to the superior noise properties and sensitivity of the imaging system. Moreover, these advances have important clinical implications, improving patient comfort, minimizing radiation exposure, and optimizing healthcare resource utilization through increased scanner throughput and reduced operational costs [34].

Several limitations of this study merit consideration. First, the single-center design and relatively small sample size —partly due to the rarity of mUM—limit the generalizability of our results. Validation in larger, multicenter cohorts is essential. However, it is noteworthy that the deep learning-based segmentation and PERCIST analysis were conducted using the RECOMIA cloud-based platform, which is publicly accessible and vendor-neutral. This enables potential adoption of our workflow by other institutions, including those without LAFOV PET/CT systems, as long as acquisition protocols and image quality are comparable. Second, the follow-up duration, while consistent with prior PET/CT studies monitoring tebentafusp treatment [35, 36], remains relatively short, largely due to the aggressive clinical course of mUM. Nonetheless, all patients underwent imaging at standardized time points before and during tebentafusp therapy, ensuring methodological consistency. Third, this analysis primarily relies on AI-supported imaging data for treatment response evaluation and prognosis, with availability of ctDNA data in only a subcohort of patients. However, this exploratory ctDNA analysis was very promising, highlighting a complementary role of molecular and imaging biomarkers in response prediction and patient stratification. A more comprehensive approach, integrating imaging with sequencing data to develop radiogenomic signatures, may enhance therapy assessment and patient stratification [37, 38], while the development of novel melanoma-specific radiopharmaceuticals could open new theranostic avenues [39]. Finally, most PET/CT findings were not histopathologically confirmed, a limitation inherent to the routine clinical practice, where such confirmation is typically not feasible.

## Conclusion

This study demonstrates the feasibility and clinical utility of AI-assisted quantification and metabolic response assessment using [1⁸F]FDG LAFOV PET/CT in patients with mUM treated with tebentafusp. Deep learning-based whole-body metabolic volumetric metrics provided prognostic insights, with higher TMTV and TLG values significantly correlating with poorer OS. Additionally, automated PERCIST response classification indicated a trend toward improved survival in patients with disease control. Importantly, although ctDNA data were available only for a subcohort of patients, dynamic changes in ctDNA levels revealed a potential prognostic role: increasing ctDNA levels showed a trend toward adverse survival outcomes and significantly higher TMTV and TLG on follow-up imaging. These findings underscore the potential of integrating advanced molecular imaging analytics into routine clinical practice to enhance risk stratification and therapeutic monitoring in this challenging malignancy.

## Data Availability

The datasets generated during and/or analysed during the current study are available from the corresponding author on reasonable request.
